# SKELETAL MUSCLE MITOCHONDRIAL RESPIRATION ASSOCIATES WITH COGNITIVE FUNCTION IN OLDER ADULTS

**DOI:** 10.1093/geroni/igae098.0791

**Published:** 2024-12-31

**Authors:** Caterina Rosano, Stephen Kritchevsky, Iva Miljkovic, Anne Newman, Peggy Cawthon, Stephen Cummings, Paul Coen

**Affiliations:** University of Pittsburgh, Pittsburgh, Pennsylvania, United States; Wake Forest University School of Medicine, Wake Forest University School of Medicine, North Carolina, United States; University of Pittsburgh, Pittsburgh, Pennsylvania, United States; University of Pittsburgh, University of Pittsburgh, Pennsylvania, United States; California Pacific Medical Center, San Francisco, California, United States; University of California San Francisco, San Francisco, Mississippi, United States; AdventHealth, Orlando, Florida, United States

## Abstract

Lower skeletal muscle oxidative capacity has been linked with poor cognition in older adults. Prior studies have relied on spectroscopy assessment of post-exercise PCr recovery rate to indirectly determine muscle oxidative capacity. Here, we evaluated if mitochondrial energetics assessed in muscle biopsies and by spectroscopy is associated with processing speed and general cognition. The sample included 858 adults (mean age 76.34 +/- 4.97 years, 59% female, 84% Non Hispanic White) from the Study of Muscle, Mobility and Aging (SOMMA). Mitochondrial respiration was measured in permeabilized fibers from biopsies of the vastus lateralis as maximal oxidative phosphorylation (MAXOXPHOS). Muscle oxidative capacity (ATPmax) was assessed by 31P MRS. Cognitive function was assessed by Digit Symbol Substitution Test (DSST), and Montreal Cognitive Assessment (MoCA) with scores adjusted for age and education, and sex. Multivariable linear regression models tested the hypothesis that higher mitochondrial energetics (MAXOXPHOS and ATPmax, in separate models) would be associated with higher scores on DSST and MoCA; all models were adjusted for race, muscle area, hypertension, diabetes and BMI. Each SD decrement in MAXOXPHOS was associated with a -1.7 point lower DSST score (95% CI: 0.68, 2.72) and a non-significantly -0.23 point lower MoCA score (95% CI: -0.00, 0.45). ATPmax was not significantly associated with either DSST or MoCA. Higher mitochondrial respiration is associated with faster processing speed in this population, independent of other known predictors of cognition. Future studies should assess the mechanisms underlying this association.

